# Supplementary material for: Determinants of Physical Activity in Older Adults: Integrating Self-Concordance into the Theory of Planned Behavior
Source: Int J Environ Res Public Health. 2021 May 27;18(11):5759. doi: 10.3390/ijerph18115759 (PMC8199322; doi:10.3390/ijerph18115759)
Supplement: Supplementary file 1 [file ijerph-18-05759-s001.zip › ijerph-1220962-supplementary.pdf]

International Journal of
Environmental Research and Public Health

MDPI

Supplementary Materials

Table S1. Quality assessment of the reflective measurement models.

Construct Indicator	Factor Loading	*t*-value	Composite Reliability	AVE
Reference Point	≥ 0.60	> 1.645	≥ 0.60	≥ 0.50
Attitude (A)			0.83	0.54
Q13A	0.69	21.415		
Q13B	0.77	33.897		
Q13C	0.70	21.153		
Q13E	0.78	34.768		
Subjective Norm (SN)			0.71	0.58
Q12A	0.45	3.748		
Q12B	0.98	39.954		
PBC (PBC)			0.80	0.67
Q11	0.87	45.627		
Q14	0.76	21.906		
Intrinsic Regulation			0.83	0.62
Q10A	0.84	56.106		
Q10E	0.83	52.436		
Q10I	0.68	23.766		
Identified Regulation			0.84	0.63
Q10C	0.80	32.388		
Q10G	0.80	33.542		
Q10K	0.79	31.211		
Introjected Regulation			0.83	0.62
Q10D	0.87	41.681		
Q10H	0.84	37.300		
Q10L	0.62	12.893		
External Regulation			0.85	0.73
Q10B	0.88	55.811		
Q10J	0.83	31.333		

Note. One item of each of the constructs' attitude (Q13D) and External Regulation (Q10F) were deleted because of insufficient factor loadings below 0.60 and because their exclusion increased the composite reliability [58].

Table S2. Differences between those with a weak and a strong habit.

	**Weak Habit (*n* = 264–284)**		**Strong Habit (*n* = 346–375)**			
	M	*SD*	*M*	*SD*	*t*	*p*
Attitude	4.32	0.60	4.87	0.26	−14.31	0.000
Subjective Norm	3.27	1.17	3.30	1.24	−0.33	0.739
PBC	4.35	0.73	4.85	0.40	−10.28	0.000
Intention	4.27	0.82	4.83	0.47	−10.42	0.000
Intrinsic	3.52	0.96	4.60	0.58	−16.66	0.000
Identified	4.50	0.86	4.81	0.43	−13.55	0.000
Introjected	2.83	1.06	2.88	1.31	−0.60	0.552
External	1.85	1.06	1.49	0.92	4.59	0.000

Table S3. Results of the hypothesis testing of the main structural model.

Target Construct	R^2		Q^2	
**Impact Direction**	**Path Coefficient**	***t*-value**	**f^2**	**Hypothesis Testing**
Intention Strength (IS)	0.313		0.303	
H1a: Attitude (+) → IS	0.135	3.661***	0.023	(√)
H2a: PBC (+) → IS	0.496	13.968***	0.305	√
H3a: Subjective Norm (o) → IS	−0.059	1.925	0.005	√
Intrinsic Regulation (INR)	0.430		0.250	
H4a: Attitude (+) → INR	0.536	15.743***	0.432	√
H5a: PBC (+) → INR	0.226	5.518***	0.076	√
H6a: Subjective Norm (−) → INR	0.008	0.244	0.000	x
Identified Regulation (IDR)	0.320		0.190	
H7a: Attitude (+) → IDR	0.390	9.470***	0.192	√
H8a: PBC (+) → IDR	0.275	6.004***	0.095	√
H9a: Subjective Norm (−) → IDR	0.081	2.609**	0.010	x
Introjected Regulation (IJR)	0.077		0.043	
H10a: Attitude (−) → IJR	0.022	0.695	0.000	x
H11a: PBC (o) → IJR	−0.023	0.669	0.000	√
H12a: Subjective Norm (+) → IJR	0.278	7.901***	0.083	√
External Regulation (EXR)	0.156		0.105	
H13a: Attitude (−) → EXR	−0.133	3.501***	0.018	(√)
H14a: PBC (o) → EXR	−0.080	2.250*	0.007	x
H15a: Subjective Norm (+) → EXR	0.361	12.262***	0.154	√

Note. Hypotheses regarding (+) positive relation, (−) negative relation, (o) no relation. */**/*** The correlation is unilaterally significant at the level of 0.05/0.01/0.001.

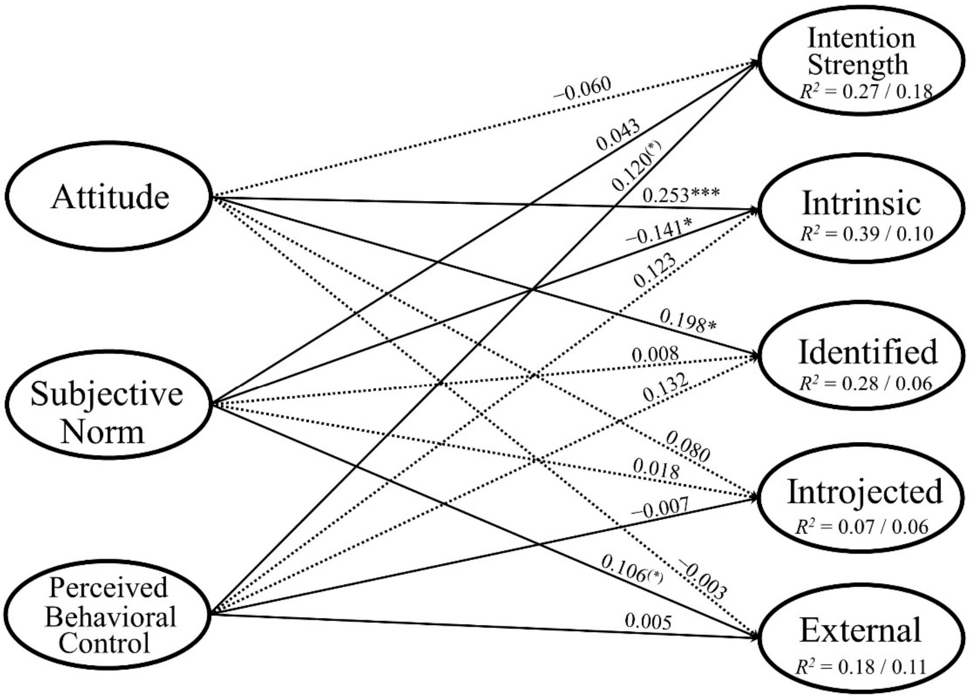

Notes. The continuous arrows illustrate the (partly) confirmed hypothesis, while the dashed arrows symbolize the rejection of a hypothesis. (*)/*/*** The path coefficient difference is unilaterally significant at the level of 0.10/ 0.05/ 0.001.

Figure S1. Results of the Group Comparison.
